# Two Groups Not on All Fours

**DOI:** 10.1371/journal.pmed.0030075

**Published:** 2006-01-31

**Authors:** Hugh Young

**Affiliations:** **1**Circumstitions.com

A prime requirement in any controlled study is that as far as possible, all conditions apart from the one being tested should be the same.

In the Auvert study, the men from the intervention group were instructed, in effect, as follows: “When you are circumcised you will be asked to have no sexual contact in the six weeks after surgery. To have sexual contact before the skin of your penis is completely healed could lead to infection if your partner is infected with a sexually transmitted disease. It could also be painful and lead to bleeding. If you desire to have sexual contact in the six weeks after surgery, despite our recommendation, it is absolutely essential that you use a condom” (Text S3 in [ 1]).

So the men in the intervention group were given very different instructions about sexual behaviour than those in the control group—in precisely the field where their risk of HIV infection was most affected. This could have differentially affected their sexual behaviour, and perhaps how they reported it. The time they spent waiting for and recovering from their surgery could also have exposed them to more safe-sex information and influence than the control group.

The control group was given no medical intervention at all. The study would have come closer to reaching equivalence between the two groups if a placebo surgery had been performed on the penis, such as opening and suturing an annular incision on the shaft, but leaving the foreskin, the supposed portal of HIV infection. The control group would then have needed identical instructions to those given to the intervention group; then, the two groups would have had much more equivalent risk.
